# N6-Methyladenosine-Sculpted Regulatory Landscape of Noncoding RNA

**DOI:** 10.3389/fonc.2021.743990

**Published:** 2021-10-15

**Authors:** Zhongyuan Zhang, Wei Wei, Hao Wang, Jiangning Dong

**Affiliations:** ^1^ Department of Radiology, The First Affiliated Hospital of the University of Science and Technology of China, Anhui Provincial Cancer Hospital, Hefei, China; ^2^ Department of Laboratory Medicine, Division of Life Sciences and Medicine, The First Affiliated Hospital of the University of Science and Technology of China, Hefei, China

**Keywords:** N^6^-methyladenosine (m6A), post-transcriptional modification, noncoding RNAs, cancer, regulation

## Abstract

The exploration of dynamic N6-methyladenosine (m6A) RNA modification in mammalian cells has attracted great interest in recent years. M6A modification plays pivotal roles in multiple biological and pathological processes, including cellular reprogramming, fertility, senescence, and tumorigenesis. In comparison with growing research unraveling the effects of m6A modifications on eukaryotic messenger RNAs, reports of the association between noncoding RNAs and m6A modification are relatively limited. Noncoding RNAs that undergo m6A modification are capable of regulating gene expression and also play an important role in epigenetic regulation. Moreover, the homeostasis of m6A modification can be affected by noncoding RNAs across a broad spectrum of biological activities. Importantly, fine-tuning and interaction between these processes are responsible for cell development, as well as the initiation and progression of the disease. Hence, in this review, we provide an account of recent developments, revealing biological interactions between noncoding RNAs and m6A modification, and discuss the potential clinical applications of interfering with m6A modification.

## 1 Introduction

Since the definition of the term “epitranscriptomics”, more than 100 types of RNA modifications have been recognized in living organisms. As an important mechanism of epitranscriptomics, first characterized in the 1970s, N6-methyladenosine (m6A) is the most frequently observed internal chemical modification in eukaryotic mRNA (1–3). As illustrated by a large body of research, m6A RNA modification modulates sophisticated RNA processes, including splicing, nucleic transport, degradation, and translation efficiency, thereby broadening the diversity of RNA modification (1–3). Preferentially found in the brain, heart, and kidney, and highly conserved between humans and mice, m6A modification mainly lies in the 3’-UTR of mRNAs, near the stop codons and within internal exons (1). m6A is a dynamic and reversible event, that is manipulated selectively by enzymes that play the part of “writers”, “erasers”, and “readers”. These effectors are equipped with multifaceted and tunable properties based on the cellular context. For certain RNA, the accessibility and biological activity of m6A modification very likely influences the outcome of physiological or pathological processes.

### 1.1 Biological Role of m6A Modification

#### 1.1.1 Writers

**Figure 1:**
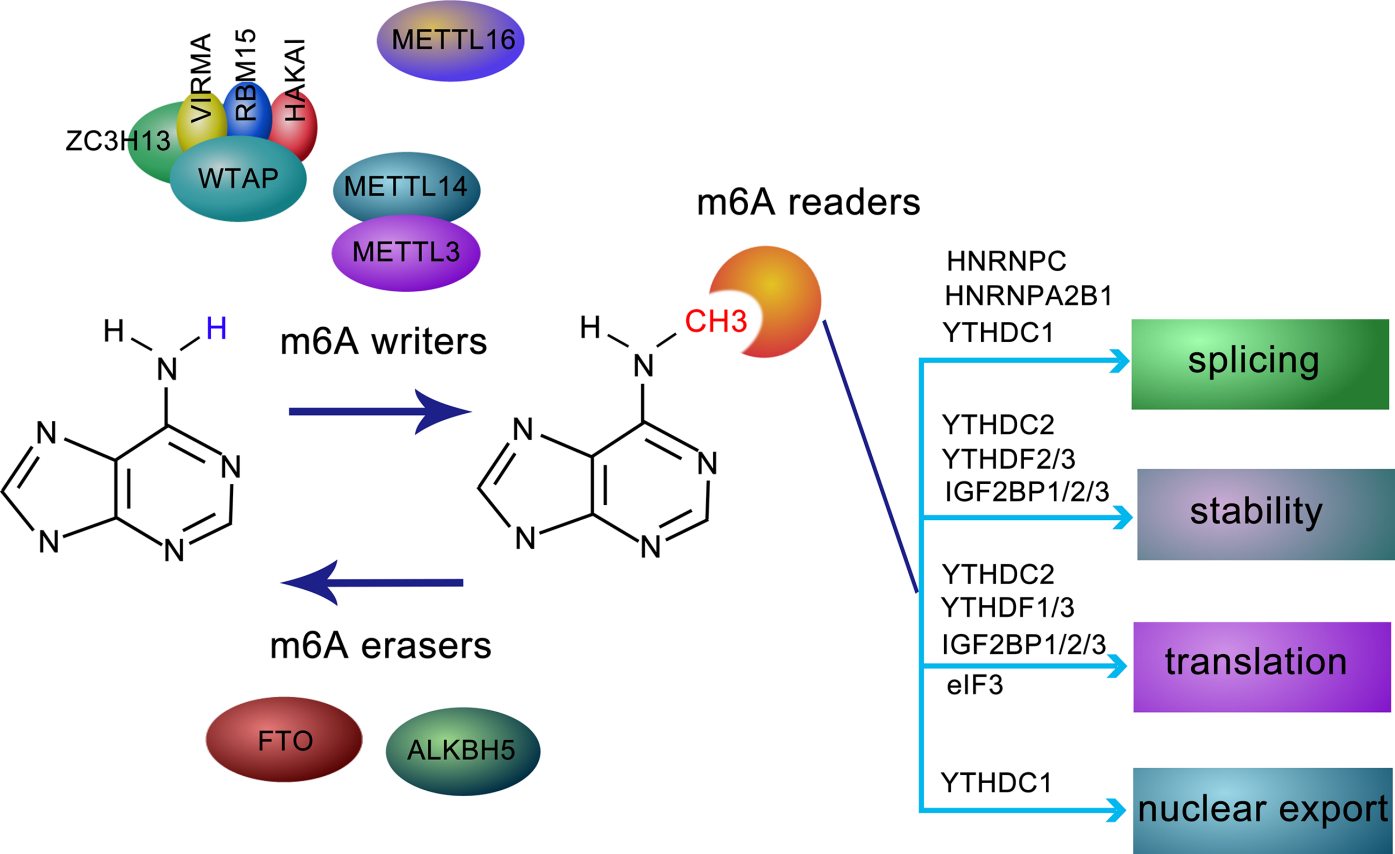
Molecular composition and regulation mechanism of m6A methylation modification. m6A methylation is a dynamic and reversible process coordinated by methyltransferases (defined as “writers”, including METTL3, METTL14, WTAP, ZC3H13, HAKAI, VIRMA, and RBM15), demethylases (defined as “erasers”, FTO and ALKBH5), and “readers”, such as YTHDF1-3, YTHDC1, IGF2BPs, HNRNPC, HNRNPA2B1, and eIF3, recognize and bind to m6A-modified RN and mediating RNA splicing, stability, translation, and RNA nuclear export.

m6A modification is regulated by a methyltransferase complex, the core subunits of which are composed of METTL3 and METTL14, as well as other auxiliary cofactors, including WTAP, VIRMA, RBM15/15B, ZC3H13, and HAKAI. METTL3, the first identified methyltransferase, often forms a heterodimer with METTL14, which functions as a conformational switch for the catalytic activity of METTL3 (2). The METTL3-METTL14 complex accurately binds to targets with the assistance of WTAP, a regulatory subunit without catalytic a domain. Interestingly, METTL3 can stimulate the translation of a set of oncogenes independently of m6A modification in lung cancer (3). VIRMA acts as an RNA-binding protein related to splicing and processing (3). The interaction of RBM15 and its paralog RBM15B with METTL3 is dependent on WTAP, both RBM15 and RBM15B bind to the U-rich sequence near the m6A sites (4). Correct nuclear localization of the writer complex relies on ZC3H13, which modulates the differentiation of mouse embryonic stem cells and nuclear RNA m6A methylation (5). HAKAI, an E3-ligase for E-cadherin, shares several targets with WTAP and is closely related to epithelial-mesenchymal transition (EMT) (6). Another effector of m6A modification, METTL16, other than being the m6A reader of U6 snRNA, also mediates atypical m6A modifications. Most of the m6A residues are found in introns (7), suggesting that METTL16 probably binds to pre-mRNAs in addition to small nuclear RNA (**Figure 1**).

#### 1.1.2 Erasers

m6A modification is reversible due to the activity of two erasers, FTO and ALKBH5, which remove m6A modification from modified RNAs. While the former is predominantly expressed in adipose tissue and hypothalamic nuclei (8) and are related to human obesity and energy metabolism (6), the latter is mainly distributed in the testis, and its depletion leads to aberrant spermatogenesis (9). Given their different localization, FTO and ALKBH5 may function separately in a tissue-specific manner (3) (**Figure 1**).

#### 1.1.3 Readers

The fate of m6A-located transcripts is determined by a series of readers. The YTH (YT521-B homology) family was the first identified class of readers recognizing sites of m6A modifications. Among these, YTHDC1 regulates splicing by recruiting splicing factors (4). While YTHDF1 and YTHDC2 elevate the translation efficiency of m6A-modified RNA, YTHDF2 and YTHDF3 prompt the decay of targeted RNAs (10). In mammalian germ cells, YTHDC2 is essential for meiosis depending on m6A modifications (11). Insulin-like growth factor 2 mRNA-binding proteins 1/2/3 (IGF2BP1/2/3), a family of newly discovered m6A readers, modulate m6A-transcripts localization, stability, and translational efficiency (12). Different from the abovementioned counterparts, FMR1 is an indirect and sequence-context-dependent m6A reader that inhibits translation (13). HNRNPA2B1, an HNRNP family protein, accelerates miRNA processing after recognizing m6A-pri-miRNA and attracting accessory microprocessors and triggers alternative splicing similar to the activity of METTLs on mRNA (14). HNRNPC is involved in the alternative splicing of m6A-transcripts indirectly. Eukaryotic initiation factor 3 (eIF3) contributes to ribosome loading by binding to m6A-modified sequences (15). Recently, it has been reported that NF-kappaB activating protein (NKAP) preferentially binds to m6A-pri-miR-25 rather than pri-miR-25 (16), indicating a role for the m6A reader NKAP in pancreatic cancer (**Figure 1**).

### 1.2 Biological Role of Noncoding RNA

The majority of human transcription products are non-coding RNAs (ncRNA) that are ubiquitously expressed in a broad spectrum of tissues (17). These RNAs engage in intricate gene expression processes, such as RNA splicing and protein translation, although they have little or no capacity for protein-coding. Recently, by virtue of the advancement in detection methods, numerous noncoding transcripts that were previously overlooked and merely regarded as intermediaries of protein synthesis, have been characterized to be critical for the posttranscriptional regulation of transcriptome expression (17). In general, ncRNAs mainly contain long noncoding RNA (lncRNA), microRNA (miRNA), circular RNA, rRNA, tRNA, and snRNA. Considering the vital role of the first three ncRNA listed above, this review describes their functions in multiple biological and pathological processes.

#### 1.2.1 Long Noncoding RNA

LncRNA, consisting of over 200 nucleotides, can be divided into five types and defined as intergenic lncRNAs, intronic lncRNAs, antisense lncRNAs, bidirectional lncRNAs, and enhancer lncRNAs, according to their genomic organization (17). LncRNAs, whose exact source remains obscure at present, execute the molecular functions as decoys, guides, scaffolds, and signals (18). Widely expressed in eukaryotes, they were previously defined as anomalies in the process of transcription. However, it has now been universally accepted that lncRNAs modulate various processes of gene transcription or post-transcription *via* interacting with mRNAs, miRNAs, or proteins (19). Approximately half of lncRNAs are believed to be retained in the nucleus and fine-tune the chromatin spatial architecture (19), or interact with the chromosome related proteins constituting the RNA-DNA complex. Moreover, recent studies have shown that some cytoplasmic lncRNAs migrate and act in ribosomes (20). Importantly, lncRNAs are very likely to act as competing endogenous RNA (ceRNA) in regulating the repression effects of miRNAs by competitively binding to miRNAs (21).

#### 1.2.2 MicroRNA

As a class of endogenous non-coding single-stranded RNA comprising 21–24 nucleotides, miRNAs function as sequence-specific negative regulators in post-transcriptional gene silencing by recognizing target mRNAs and mediating mRNA cleavage or translational repression (22). miRNAs are produced from primary miRNAs (pri-miRNAs), which differ in length and are located in the introns of host genes. Pri-miRNAs are first converted into hairpin-shaped precursor microRNA (pre-miRNA) upon the cleavage of Drosha (23). Afterward, pre-miRNAs translocate from the nucleus to the cytoplasm where Dicer digests pre-miRNA to generate functionally mature miRNA (24). The nucleotides 2-8 at the 5’-end of miRNAs decide the binding sequence of mRNA, hundreds of mRNAs can be targeted by a single miRNA, and vice versa, each mRNA can also be subjected to regulation by different miRNAs simultaneously or sequentially, this phenomenon may be explained by the imperfect base-pairing between miRNAs and mRNAs, and because most binding sites are positioned within the 3’-UTR of mRNAs (25).

#### 1.2.3 Circular RNA

Circular RNA (circRNA), characterized by the lack of free 5’ and 3’ ends, is a novel class of ncRNAs generated from non-canonical back-splicing or the exon skipping of linear pre-mRNAs (26). CircRNAs are mostly composed of internal exons and reside predominantly in cytoplasm. Intriguingly, compared to their linear counterparts, the covalently closed loop structure endows circRNAs with preferable stability (27).

The degradation of circRNAs is solely dependent on endoribonucleolytic cleavage owing to the absence of the 5’ cap and 3’ poly(A) tail (18). The resistance to RNA exonuclease or RNase R hinders their degradation and enhances their detectability. Another property of circRNAs lies in their specific expression in certain cell types and tissues. In addition, circRNAs are sensitive to cellular stress. All these features render the potential of circRNAs to be novel molecular biomarkers for the diagnosis of diseases (4).

## 2 LncRNA and N6-Methyladenosine

**Figure 2:**
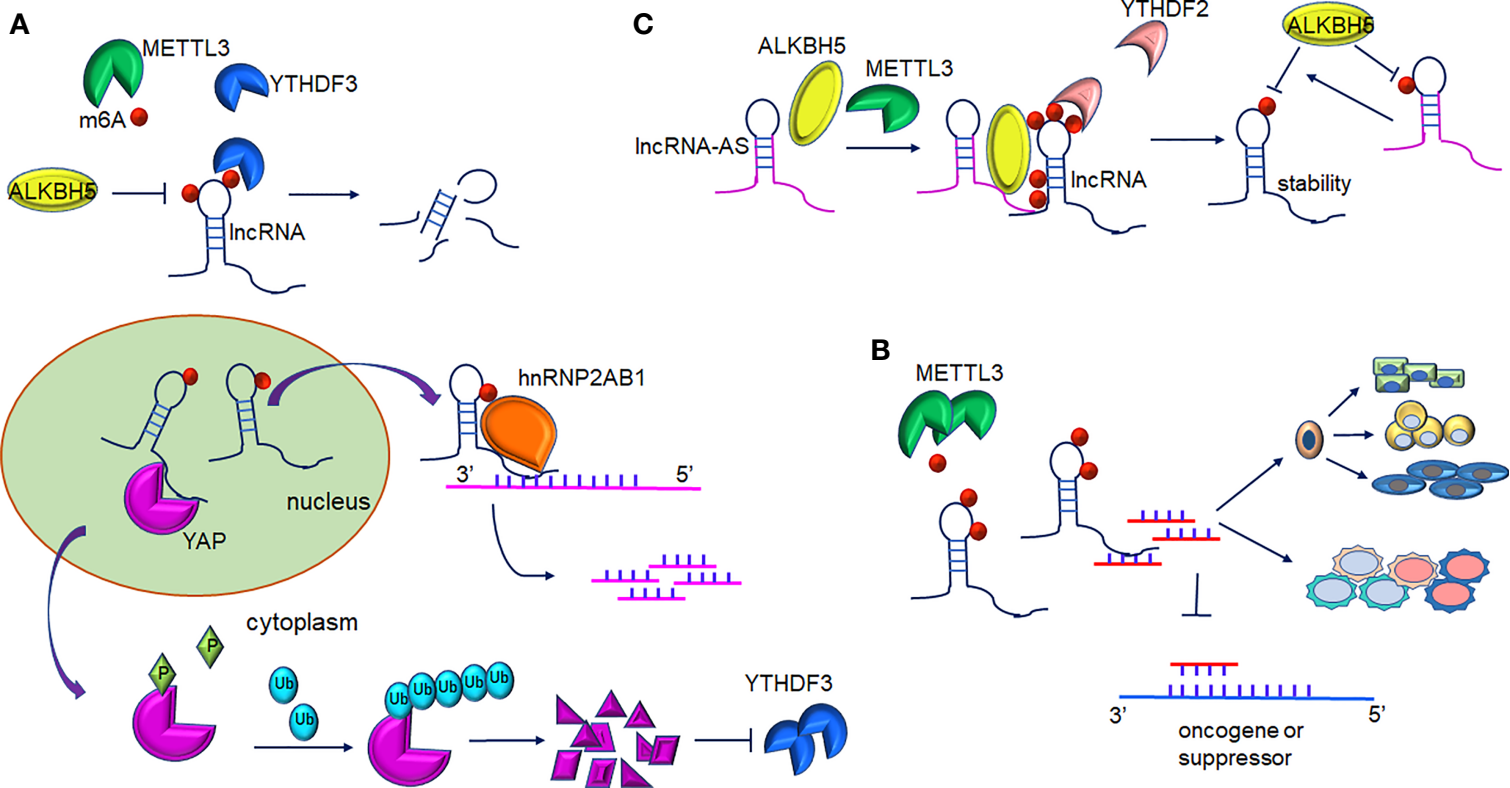
Interplay between lncRNAs and m6A modification. **(A)** m6A-mediated lncRNAs target mRNA/protein. m6A-lncRNA GAS5 targets endogenous YAP, which induces YAP degradation and downregulation of its target YTHDF3. In turn, YTHDF3 promotes degradation of m6A-modified GAS5. m6A-lncRNA RP11 binds to the mRNA of FBXO45 and SIAH1 promoting their degradation. **(B)** m6A-mediated methylation of lncRNA-miRNAs activity in gene expression. METTL3-mediated m6A-lncRNAs methylation serves as a sponge to restrain miRNA activity and thus abolishes miRNAs-modulated mRNA inhibition. Furthermore, interaction of lncRNA and miRNA contribute to proper differentiation of mouse embryonic stem cells and the inflammatory response of cells. **(C)** The role of LncRNA-AS in m6A-mediated regulation. LncRNA can directly interact with ALKBH5 or METTL3. GAS5-AS binds to m6A-GAS in a YTHDF2-dependent manner and reduces its stability. Furthermore, demethylation of GAS5 is mediated by ALKBH5 and GAS-AS facilitate this process. ARHGAP5-AS1 can recruit METTL3 to stimulate the m6A modification of ARHGAP5 and increase its stabilization in the cytoplasm.

Similar to mRNAs, lncRNAs are subjected to m6A methylation in various cell lines. m6A residues show a preference for locating in lncRNA transcripts that have been subjected to alternative splicing (19), indicating that m6A deposition may play a potential role in the formation of lncRNA isoforms. m6A methylation sites are well-distributed along transcripts, in comparison to mRNAs modification patterns (27). As illustrated by recent studies, the abundance of m6A-modified lncRNAs dramatically decreases in human fetal tissues (21), in comparison with mRNA (**Figure 2** and **Table 1**).

**Table 1:** LncRNAs in disease or normal cells and related m6A effectors.

LncRNA	Cancer	Expression	Pathway	m6A effector	Reference
RP11	CRC	Upregulation	ZEB1	hnRNPA2B1 METTL3 ALKBH5	(19)
GAS5	CRC	Downregulation	YAP	YTHDF3	(28)
NEAT1	GC	Upregulation	EZH2	ALKBH5	(29)
PVT1	OS	Upregulation	—	ALKBH5 YTHDF2	(30)
XIST	human cells	—	—	METTL3, YTHDC1 RBM15/15B/WTAP	(4)
MALAT1	mESC	—	—	METTL16	(7)
DANCER	PC	—	—	IGF2BP2	(31)
FAM225A	NPC	Upregulation	ITGB3/FAK/PI3K/AKT	METTL3	(32)
Linc1281	mESC	—	Lin28	METTL3	(33)
Linc00958	HCC	Upregulation	SREBP1/FASN/SCD1/ACC1/HDGF	METTL3	(34)
MEG3	HCC	Downregulation	BTG2	METTL3	(35)
PCAT6	PCa	Upregulation	IGF1R	METTL3	(36)
XIST	OPLL	Upregulation	USP8	METTL3	(37)
Linc00470	GC	Upregulation	PTEN	METTL3 YTHDF2	(38)
BLACAT2	GC	Upregulation	—	METTL3	(39)
Olfr29-ps1	MDSCs	Upregulation	IL-6/MyD88	METTL3	(40)
LINC00958	BC	Upregulation	YY1	METTL3	(41)
GAS5-AS1	CC	Downregulation	—	YTHDF2, ALKBH5	(42)
FOXM1-AS	GBM	Upregulation	SOX2	ALKBH5	(43)
KCNK15-AS1	PC	Downregulation	—	ALKBH5	(44)
ABHD11-AS1	NSCLC	Upregulation	—	METTL3	(45)
ARHGAP5-AS1	GC	Upregulation	SQSTM1	METTL3	(46)
HOTAIR	NSCLC	—	—	m6A signal	(47)

CRC, colorectal cancer; GC, gastric cancer; OS, osteosarcoma; mESC, mouse embryonic stem cells; PC, pancreatic cancer; NPC, nasopharyngeal carcinoma; HCC, hepatocellular carcinoma; PCa, prostate cancer; OPLL, ossification of the posterior longitudinal ligament; GC, gastric cancer; MDSCs, myeloid-derived suppressor cells; BC, breast cancer; CC, cervical cancer; GBM, glioblastoma; PC, pancreatic cancer; NSCLC, non-small cell lung cancer.

### 2.1 m6A-Mediated Regulation on LncRNA Expression

The dysregulation of lncRNAs has been proven to play a non-negligible role in tumorigenesis. In colorectal cancer (CRC) cells, increased expression of lncRNA RP11 in the nucleus and chromatin is mediated by m6A methylation. RP11 is capable of directly binding to the mRNA of Siah1 and Fbxo45 and then stimulates mRNA degradation, leading to the post-translational stabilization of ZEB1 (19). As revealed by Ni et al., m6A methylation participates in the regulation of YAP signaling in the progression of CRC. lncRNA GAS5 inhibits CRC progression *via* mediating the phosphorylation and degradation of YAP. However, the interaction of GAS5 and the m6A reader YTHDF3 results in the degradation of the former, contributing to the suppression of cell proliferation and metastasis abilities (28) (**Figure 2A**). It is of note that lncRNA can also interact with m6A writers such as METTL3. In gastric cancer, LINC00470 associates with METTL3 to weaken the stabilization of PTEN mRNA. YTHDF2 accounts for the detection of m6A sites within PTEN mRNA (38). In addition, lncRNA NEAT1 is demethylated by ALKBH5, consequently upregulates and prompts the invasion and metastasis of GC cells (29). In addition, In osteosarcoma, m6A demethylation on lncRNA PVT1 is mediated by ALKBH5 and associated with malignant properties (30).

Patil et al. demonstrated that the knockdown of METTL3 impaired the silencing of gene transcription in human cells. This process was modulated by lncRNA XIST (4). An enhanced abundance of m6A modification on XIST residues is mediated by RBM15/15B, which recruits the WTAP-METTL3 complex, and subsequently, YTHDC1 preferentially binds to m6A residues on XIST. Methyltransferase METTL16 can interact with lncRNA MALAT1, a cancer-related lncRNA (7, 48, 49). The crosslinking sites mainly occur at the 3’ UTR of MALAT1, wherein the triple helix element is usually recruited. m6A methylation disrupts the local RNA architecture of MALAT1 (50) and impedes the binding of RBPs to MALAT1. The aberrant expression of IGF2BP2 has been associated with insulin resistance, diabetes, and even neoplasia (51). IGF2BP2 interacted with lncRNA DANCR modified by m6A, resulting in the stability enhancement of DANCR. IGF2BP2 and DANCR jointly contribute to the proliferation and stemness-like properties of pancreatic cancer cells (31).

### 2.2 m6A-Mediated LncRNA-miRNA Interaction

Based on genome-wide microarray analysis, a novel upregulated oncogenic lncRNA FAM225A was found to be significantly related to recurrence and distant metastasis in nasopharyngeal carcinogenesis (NPC) (32). Enhanced stabilization of FAM225A likely resulted from the enrichment of m6A modifications within its transcripts. Most FAM225A is located in the cytoplasm and absorbs miR-590-3p and miR-1275, resulting in the upregulation of their common target integrin β3(ITGB3). ITGB3 was shown to account for malignant phenotype progression in NPC cells (32). Yang et al. found that lncRNA 1281 could sequester pluripotency-related let-7 family miRNAs in mouse embryonic stem cells (mESC) (33). Sufficient m6A modification on lncRNA 1281 transcripts was necessary for this direct RNA-RNA interaction, which maintained mESC markers and proper differentiation (**Figure 2B** and **Table 1**). Studies have revealed that lncRNA 00958 acts as an oncogenic gene in gliomagenesis (52), whereas its role in hepatocellular carcinoma (HCC) was not revealed until very recently. Zuo et al. disclosed that METTL3 induced the upregulation of lncRNA 00958, which exerted lipogenesis and the unfavorable survival of HCC patients (34). LncRNA 00958 could sponge miR-3619-5p whose target was hepatoma-derived growth factor (HDGF). The inhibitory effects of miR-3619-5p in the dissemination and invasion of HCC cells have also been described previously (53). In addition, m6A modification is also involved in nontumor pathologic or physiological processes, including heart and brain ischemia reperfusion injury (54). LncRNA MALAT1 targets miR-26b in the regulation of PTGS2, and has been reported to exacerbate inflammatory response in myocardial ischemia infarction patients, while m6A modification may also enhance MALAT1 expression (55). The lncRNA-BLACAT2 is a sponge of miR-193b-5p and has been associated with the progression of gastric cancer while silencing BLACAT2 inhibited cancer cells migration and invasion. A further study showed that METTL3 was a direct target of miR-193b-5p (39). Furthermore, similar to ceRNA, the lncRNA pseudogene Olfr29-ps1 sponged miR-214-3p and influenced IL6-mediated m6A modification, which jointly modulated the differentiation of myeloid-derived suppressor cells (40). Likewise, METTL3-mediated LINC00958 upregulation also played a similar ceRNA role over miR-378a-3p to promote YY1 expression in BC tumorigenesis (41). METTL3 induced LncRNA MEG3 stability and suppressed the progression of HCC by targeting miR-544b/BTG2 signaling (35). Furthermore, m6A methylation on lncRNA PCAT6 contributed to PCAT6 upregulation in an IGF2BP2‐dependent manner in prostate cancer (36). Yuan et al. also showed that METTL3 promoted osteogenic ossification through the upregulation of lncRNA XIST, and further investigation confirmed that lncRNA XIST regulated osteogenic differentiation of primary ligament fibroblasts *via* miR-302a-3p, which targets ubiquitin-specific protease 8 (USP8) (37).

### 2.3 m6A-Methylation of LncRNA-Antisense ncRNA

A recent study showed that GAS5-AS1, the antisense RNA of GAS5, was downregulated in HCC (56) and non-small cell lung carcinoma (NSCLC) (57). In cervical cancer (CC) cells, the lncRNA GAS5-AS1 attenuates m6A modification of GAS5 through antisense pairing with the GAS5 3’ UTR, and thus, epigenetically enhanced GAS5 stability in a YTHDF2-dependent fashion. Whereas knockdown GAS5-AS1 resulted in larger tumors, enhanced metastasis, and advanced prognosis in CC patients (42). The lncRNA GAS5-AS1 also interacts with ALKBH5 to induce GAS5 upregulation (**Figure 2C** and **Table 1**). Analogously, the lncRNA ARHGAP5-AS1 can recruit METTL3 to methylate ARHGAP5 mRNA in gastric cancer cells, thus, increased levels of m6A-ARHGAP5 were predictive of enhanced chemoresistance and poor prognosis (46). Zhang et al. revealed that FOXM1 maintained self-renewal and tumorigenic properties of glioblastoma stem-like cells (GSCs) (43). ALKBH5-mediated demethylation of nascent FOXM1 transcripts and upregulation of FOXM1 expression was facilitated by lncRNA FOXM1-AS. GSCs properties could be destroyed significantly when ALKBH5 or FOXM1-AS expression was blocked. He et al. determined that lncRNA KCNK15-AS1 was significantly decreased in cancer cells compared to normal pancreatic ductal epithelial cells (44) and its expression was correlated with the degree of m6A methylation. Simultaneously, ALKBH5 could demethylate KCNK15-AS1 and enhance the repression effects of KCNK15-AS1 on cancer cell viability. Furthermore, METTL3 was found to be responsible for the m6A methylation on the ABHD11-AS1 transcript and enhanced its stability in NSCLC tumorigenesis (45).

Recently, the lncRNA HOTAIR was reported to act as a plasma-derived biomarker of NSCLC, and m6A methylation was found to be co-expressed with HOTAIR (47). Moreover, many other lncRNAs, such as ANRIL, NEAT1, PVT1, TUG1, and DICER1-AS1, probably undergo m6A modification (58). These findings were conducive to improving understanding of the underlying mechanisms of human diseases and designing applicable therapeutic strategies.

## 3 MicroRNA and N6-Methyladenosine

### 3.1 m6A-Mediated Processing of Primary-miRNAs

Many studies have reported that miRNAs are abnormally expressed in different pathological processes, including diabetes, neurodegenerative diseases, and carcinomas. Pri-miRNA transcript can be methylated by METTL3 in the nucleus, and contribute to the recognition of pri-miRNAs by the microprocessor protein DGCR8 (59). Consistently, METTL3 deficiency can markedly decrease the expression of mature miRNAs. In bladder cancer, for example, upregulation of METTL3 accelerated the processing of pri-miR221/222, which promoted the proliferation, migration, and invasion of cancer cells in an m6A-dependent manner (60). Mature miR221/222 was also confirmed to have a carcinogenic role in other carcinomas (61), such as prostate cancer (62) and thyroid cancer (63). Analogously to its impact on bladder carcinoma, METTL3 facilitated the pri-miR-1246 maturation process and paved the way for the enhanced metastasis of CRC cells *via* the MAPK signaling pathway (64). Analogously, METTL3 upregulated miR-1246 expression and contributed to NSCLC cell growth (65). Likewise, miR-25-3p maturation was impeded by m6A modification mediated by METTL3 in pancreatic ductal adenocarcinoma (16). Furthermore, METTL3 drove the development of obstructive renal fibrosis by promoting miR-21-5p maturation (66). *In vitro*, the knockdown of METTL3 reduced the expression of miR-221-3p *via* m6A methylation of pri-miR-221-3p mRNA (67). It is of note that cigarette smoking has been shown to stimulate the METTL3 promoter resulting in its enhanced energetic transcription. Wang et al. provided evidence that METTL3-mediated miR-143-3p upregulation enhanced VASH1 repression, which thereafter triggered brain metastasis and the angiogenesis of lung cancer (68). Consistently, miR-873-5p maturation induced by exogenous METTL3 activity could protect mouse renal tubular epithelial cells (mRTECs) against colistin-induced nephrotoxicity (69). Interestingly, Yang et al. suggested that miR24-2 not only indirectly facilitated METTL3 transcription, but also enhanced miR6079 expression by promoting m6A methylation on pri-miR6079 in liver cancer cells (70).

**Figure 3:**
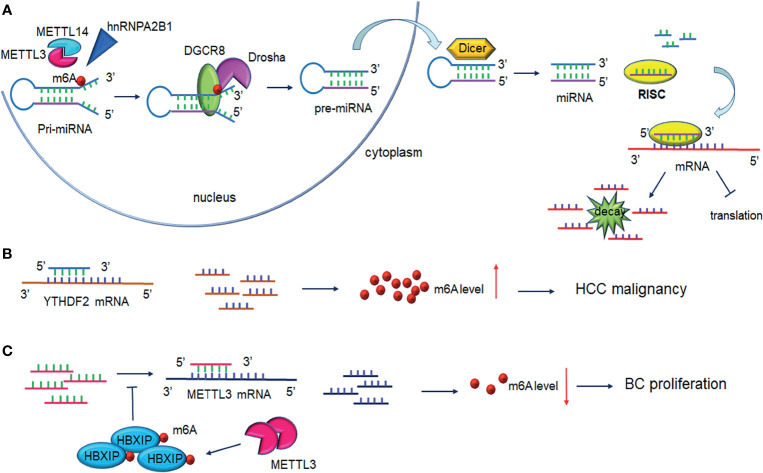
Interplay between microRNA and m6A modification. **(A)** m6A modification promotes pri-miRNAs processing. METTL3/ETTL14 facilitates DGCR8 recognition of pri-miRNAs contributing to the increased levels of mature miRNAs under the cleavage of Drosha and Dicer. One strand of the miRNAs is loaded on RISC and mediates mRNA silencing or translation repression. **(B)** MiRNAs regulate m6A levels through binding to m6A readers. MiRNAs bind to YTHDF2 mRNA inducing its degradation and interfere with HCC cell malignancy. **(C)** MiRNAs regulate m6A levels by binding to m6A writers. miRNAs degrade METTL3 mRNA and lead to decreased breast cancer cell proliferation. Upregulation of METTL3 contributes to HBXIP m6A modification, in turn, HBXIP reverses the miRNAs-induced degradation of METTL3, forming the positive feedback of METTL3/HBXIP/miRNA/METTL3. DGCR8, DiGeorge syndrome critical region 8; RISC, RNA-induced silencing complex.

Downregulation of the methyltransferase METTL14 was shown to be responsible for the aberrant m6A modification observed in HCC metastasis. miR126 is a downstream target of METTL14 and was markedly decreased in cancer tissues compared to the adjacent tissue (71). Consistently, Chen et al. revealed that downregulation METTL14 could induce pri-miRNA-375 processing arrest and reduced overall levels of miRNA-375 in CRC (72). Thus, miRNA-375 was revealed as an anti-oncogene regulated by METTL14 through the YAP/SP1 pathway and m6A methylation. miR-200a is also subject to METTL14 modulation (**Figure 3A** and **Table 2**). As a reader of the m6A label in pri-miRNAs, HNRNPA2/B1 widely directed the maturation of pri-miRNA in LCC9 breast cancer cells (83). This processing was m6A dependent and promoted endocrine resistance in LCC9 cells, resulting in the poor survival of patients with advanced tumors.

**Table 2:** MicroRNAs in disease and related m6A effectors.

MiRNA	Disease	Expression	Pathway	m6A Effector	Reference
miR221/222	BCA	Upregulation	PETN	METTL3	(60)
miR-1246	CRC	Upregulation	SPRED2, Raf/MEK/ERK	METTL3	(64)
miR-1246	NSCLC	Upregulation	PEG3	METTL3	(65)
miR-221-3p	BC	Upregulation	HIPK2/Che-1	METTL3	(67)
miR-143-3p	NSCLC	Upregulation	VASH1/VEGFA	METTL3	(68)
miR-21-5p	ORF	Upregulation	SPRY1/ERK/NF-kB	METTL3	(66)
miR-873-5p	mRTECs	—	Keap1/Nrf2	METTL3	(69)
miR126	HCC	Downregulation	—	METTL14	(71)
miRNA-375	CRC	Downregulation	YAP/SP1	METTL14	(72)
miR-25-3p	PC	Upregulation	PHLPP2/AKT-p70S6K	METTL3 NKAP	(16)
miR-145	HCC	Downregulation	—	YTHDF2	(73)
miR-4729	Hemorrhoids	Downregulation	TIE1/VEGFA	METTL14	(74)
miR-103-3p	Osteoporosis	Upregulation	—	METTL14	(75)
miR-186	HB	Downregulation	Wnt/β-catenin	METTL3, YTHDF2	(76)
miR-338-5p	GC	Downregulation	EED/CDCP1	METTL3	(77)
miR-4443	NSCLC	Upregulation	FSP1	METTL3	(78)
miR-24-2/ miR6079	HCC	Upregulation	Pim1/JMJD2A	METTL3	(70)
miR-33a	NSCLC	Downregulation	EGFR	METTL3	(79)
miR-600	NSCLC	Downregulation	EGFR/PI3K/AKT β-catenin	METTL3	(80)
miR-29a	GBM	Downregulation	QKI-6/EGFR ERK/PI3K/AKT	WTAP	(81)
miR-let-7g	BC	Downregulation	HBXIP	METTL3	(82)

BCA, bladder cancer; mRTECs, mouse renal tubular epithelial cells; HB, hepatoblastoma; BC, breast cancer. ORF, obstructive renal fibrosis.

### 3.2 miRNAs Directly Target m6A Effectors

The transcriptional regulator SRF is regulated epigenetically and responsible for cells contractility and proliferation (84). A recent study indicated that IGF2BP1 promotes the expression of SRF in a conserved and m6A-dependent manner, IGF2BP1 can impair the miRNA-directed decay of the SRF mRNA, resulting in enhanced SRF-dependent transcriptional activity (85). A sequence pairing mechanism contributes to miRNAs regulation of m6A methylation of mRNAs. Yang et al. reported that miRNA-145 expression might be considered a negative prognostic marker in liver cancer. The 3′UTR region of YTHDF2 mRNA contains direct binding sites for miR-145, and the overexpression of mir-145 increased m6A deposition and downregulated YTHDF2 expression (**Figure 3B** and **Table 2**) (73).

In hemorrhoid vascular endothelial cells, a decrease in miR-4729 expression was confirmed to be responsible for vascular cell proliferation. miR-4729 was responsible for silencing METTL14 expression, reducing TIE1 mRNA stability, and inhibiting angiogenesis (74). Additionally, miR-103-3p directly targets METTL14 to inhibit osteoblast activity (75). METTL3 has also been reported to be a target of miR-186 in hepatoblastoma (76). Overexpression of miR-186 drastically ameliorated the metastatic phenotype induced by METTL3 upregulation. Moreover, as demonstrated in bladder cancer and CRC, the oncogenic role of METTL3 substantially relied on the degradation of tumor suppressor mRNAs targeted by miRNAs. Du et al. confirmed that in NSCLC cells, miR-33a bound to the 3’-UTR of METTL3 mRNA, and thus, resulted in a decrease in m6A deposition and proliferation of cancer cells accompanied by the downregulation of METTL3 (79). Moreover, in gastric cancer, miR-338-5p has also been reported to target METTL3 and repress the m6A-mediated translation of CDCP1 (77). Furthermore, Wei et al. indicated that miR-600 repressed METTL3 expression and eliminated the oncogenic activity induced by METTL3 on NSCLC progression (80). MiR-4443 directly targeted METTL3 and regulated the expression of FSP1 *via* m6A methylation (78). It has also been reported that WTAP downregulation in GSCs was caused by miR-29a upregulation, which could interfere with the malignant potential of GSCs (81). Cai et al. demonstrated that in breast cancer cells, HBXIP increased METTL3 expression by interfering with let-7g, a miRNA binding to the 3′UTR of METTL3, which subsequently interfered with METTL3 expression (82). Interestingly, METTL3 also upregulated HBXIP expression following the marked increase of overall m6A methylation, favoring the positive feedback loop HBXIP/let-7g/METTL3/HBXIP, which strongly promoted tumor cell growth and metastasis (**Figure 3C** and **Table 2**) (82).

DDX3, a protein of the DEAD-box RNA helicases family, is involved in miRNAs demethylation due to its interaction with AGO2 protein and is involved in miRNA synthesis and function (86). Furthermore, m6A-AGO2 transcripts influence cellular miRNA levels and induced cell senescence (87). The role of miRNA in spermatogenesis was strongly evidenced in the mouse testis (88). ALKBH5 deficiency caused aberrant mammalian spermatogenesis or apoptosis because of the removal of the m6A modification on mRNAs (9) (**Figures 3B, C** and **Table 2**).

## 4 CircRNA and N6-Methyladenosine

**Figure 4:**
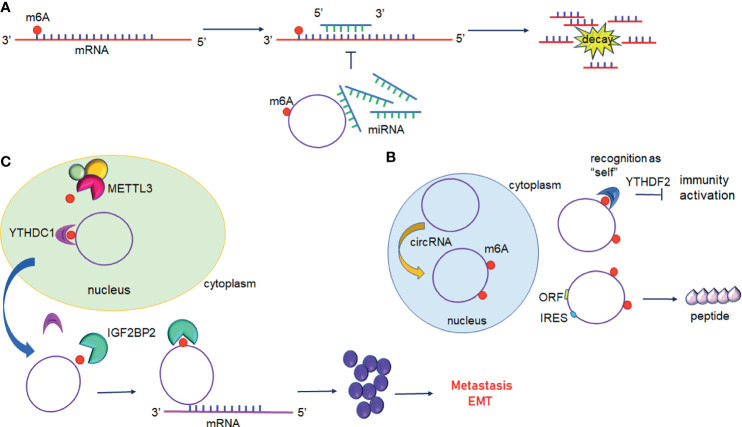
Interplay between circular RNAs and m6A modification. **(A)** circRNAs act as ceRNA to regulate gene expression. m6A modified circRNAs sequester miRNAs resulting in inhibition of miRNA-direct gene silencing. **(B)** m6A modification of circRNAs suppresses immune genes expression or initiates translation. **(C)** m6A modified circRNAs regulate gene expression by directly targeting mRNA.YTHDC1 promotes the nuclear export of circRNAs to the cytoplasm following methylation by METTL3. IGF2BP2 promotes the stability of m6A-circRNAs and interacts with oncogenic mRNAs.

In recent research, circRNAs have ceased to be considered irrelevant artefacts of splicing errors, and are considered factors impacting the post-transcriptional regulation of gene expression These single-stranded RNAs can be detected in plasma, exosomes, and cell-free saliva (89). Mounting evidence has shown that m6A modification may participate in regulating the biological functions of circRNAs. circRNA may be a potential target for cancer therapy (**Figure 4** and **Table 3**). 

**Table 3:** CircRNAs in cancer or normal cells and related m6A effectors.

Circ RNA	Cancer	Expression	Pathway	m6A effector	Reference
circRNA_104075	HCC	Upregulation	HNF4a YAP	—	(90)
circRNA-SORE	HCC	Upregulation	β-catenin	METTL3	(91)
Circ FOREIGN	Mammalian cells	—	RIG-I K63-Ub_n_	YTHDF2 METTL3	(92)
Circ NSUN2	CRC	Upregulation	HMGA2	YTHDC1 IGF2BP2	(93)
circCUX1	HPSCC	Upregulation	Caspase1	METTL3	(94)
Circ PVRL3	GC	Downregulation	EIF4A3	—	(95)
Circ E7	CC	—	HPVs	—	(96)
circNDUFB2	NSCLC	Downregulation	TRIM25	IGF2BPs	(97)
circSTAG1	MDD	Downregulation	FAAH	ALKBH5	(98)
circMAPK4	HCC	Upregulation	miR-139	YTHDF1	(99)
circPTPRA	BC	Downregulation	MYC/FSCN1	IGF2BP1	(100)
**Others**					
28S rRNA	HCC	—	—	ZCCHC4	(101)
U6 snRNA	—	—	—	METTL16	(7, 102, 103)

HPSCC, Hypopharyngeal squamous cell carcinoma; MDD, major depressive disorder.

### 4.1 m6A-Regulated miRNA/RBP Sponges of circRNA

In addition to mRNAs, pseudogenic RNAs, or lncRNAs, circRNAs have also be defined as ceRNA and compete with miRNAs or RBPs (**Figure 4A**) (104). These competing transcripts probably cross-regulate each other. In primary liver cancer, the highly expressed circRNA_104075 stimulated YAP expression both at the mRNA and protein levels (90). Further study has suggested that removal of the m6A modification on the 3′UTR region of YAP was essential for miR-582-3p binding to YAP. Furthermore, serum circ_104075 possessed high sensitivity and specificity for HCC diagnosis. circRNA-SORE sustains sorafenib resistance in HCC, and m6A modification increases the stability of circRNA-SORE (91). Yang et al. characterized eleven m6A containing circRNAs, among which seven circRNAs were significantly correlated with YTHDF2 (105). Furthermore, Chen et al. demonstrated that circFOREIGN can potentially activate gene expression of immunogenic factors (92). Nevertheless, YTHDF2 abrogated innate immunity by sequestering m6A-circRNA (**Figure 4B** and **Table 3**). The study established a patient-derived xenograft (PDX) tumor model for CRC and found that circNSUN2 strongly interfered with liver metastasis of CRC (93). The exon5-exon4 junction site of circNSUN2 was identified as the m6A modification site. The binding of YTHDC1 to the m6A-modified circNSUN2 promoted a complex nuclear to cytoplasmic export analogous to mRNA trafficking (**Figure 4C** and **Table 3**) (93). Interestingly, Wu et al. showed that METTL3 stabilized the expression of circCUX1 through the m6A methylation modification, which in turn, promoted radiotherapy resistance of hypopharyngeal cancer *via* caspase1 (94).

### 4.2 m6A-Mediated Translation Potential of circRNA

Some circRNAs carrying internal ribosome entry sites (IRESs) have the potential capacity of coding proteins (106). Granados-Riveron reported that a single m6A modification was sufficient to instigate the functional translation of circRNAs in the presence of eIF4G2 and YTHDF3 (107). Sun et al. found that circPVRL3 structure contained RRm6ACH (R = G or A; H = A, C or U), ORF, and IRES (95). Previous studies have documented that the m6A modification could regulate circRNA translation (108). Thus, it is reasonable to believe that circPVRL3 could translate into detectable peptides endogenously under certain cellular stresses. Zhao et al. reported that circE7 is found mainly in the cytoplasm and derives from oncogenic human papillomaviruses (HPVs) (96). CircE7 is highly m6A modified and translates to E7 oncoprotein following its association with polysomes.

### 4.3 circRNAs-Mediated Regulation on m6A Effectors

circRNAs also play a vital role in regulating the expression of m6A-related proteins. It has been reported that circNDUFB2 is downregulated in NSCLC and inhibits the malignant progression of NSCLC (97). Mechanically, circNDUFB2 was found co-localized with IGF2BPs in the cytoplasm and physically interacted with IGF2BP1/2/3. Interestingly, overexpression of circNDUFB2 has no effect on the mRNA levels but protein levels of IGF2BPs. Further research found that circNDUFB2 reduces IGF2BPs stability *via* ubiquitin/proteasome-mediated degradation of IGF2BPs (97). circSTAG1 can bind with ALKBH5 in the astrocyte cytoplasm of the chronic unpredictable stress-treated mouse hippocampus, overexpressed circSTAG1 absorbs ALKBH5 and decreases the translocation of ALKBH5 into the nucleus, resulting in enhancement of m6A methylation on fatty acid amide hydrolase (FAAH) mRNA and subsequent degradation of FAAH (98). In addition, circMAPK4 acts as a ham-miR-139-5p sponge to regulate the expression and activity of YTHDF1 (99). circPTPRA can directly bind to the KH3 and KH4 domains of IGF2BP1, and block recognition of m6A-modified MYC and FSCN1 transcripts in BC cells (100).

## 5 Other Noncoding RNA and m6A

m6A modification may also occur on rRNA and spliceosome RNA, albeit the relevant mechanism is not as well-known as that of mRNA or the abovementioned ncRNA. Ma et al. reported that human 28S rRNA undergoes m6A modification at position A4220 by ZCCHC4, which is aberrantly expressed in tumor tissues and positively regulates HCC cancer cell proliferation (101). An additional rRNA m6A modification is located on A1832 in 18s rRNA. These rRNAs m6A-modifications influence many aspects of ribosome activity, including localization, tertiary structure, and dynamics (109). In contrast to METTL3-METTL14-mediated m6A modification, METTL16 modified sites are primarily located in either introns or exon-intron boundaries (49). METTL16 is responsible for the methylation of MAT2A mRNA and the spliceosome U6 snRNA (49, 102, 103, 110). U6 snRNA is crucial to pre-mRNAs splicing events. U6 snRNA carries m6A modification at position A43, an evolutionarily conserved nucleotide, and is lethal if mutated in yeast models (111).

## 6 Perspectives

In recent years, the emergence of transcriptomic approaches and bioinformatics have placed m6A modification to the research spotlight, and have expanded the full scope of m6A targets, including coding RNA and ncRNAs, and have convincingly elucidated their roles in multiple mammalian cell types. METTL16, a newfound m6A effector, acts as both a writer and U6 snRNA reader (112). In addition, METTL16 modified sites almost lie within introns, which is different from that of METTL3-METTL14 mediated sites, indicating a novel specific mechanism in methylation modifications. The dual identity of METTL16 deserves further exploration. During oxidative stress, NSUN2-mediated 5-methylcytosine (m5C) activity together with METTL3-METTL14-mediated m6A methylation contributes to synergistic upregulation of p21 (113). Methylation at m6A can facilitate the methylation at m5C, and vice versa. This functional interconnection between methylation sites leads to doubt about whether other chemical modifications can promote or inhibit coding RNA or ncRNAs at the posttranscriptional level.

In particular, m6A distribution mapping in lncRNAs is distinct compared to other mRNAs, but underlying effects remain currently unknown. Many lncRNAs are retained in the nucleus, which implies they exert their regulatory effects on the chromatin; however, this effect also can be impaired by m6A deposition. What are the effects of lncRNAs and m6A joint modulation of chromosomal conformations? Functionally, m6A-sculpted lncRNAs levels in the fetus are fewer than the mRNA counterparts, suggesting m6A-lncRNAs may be related to cell senescence in biological development. lncRNAs are expressed in a cell-type-dependent and tissue-specific-dependent manner. This pattern is conducive to the identification of novel landmarks for certain diseases, but also raises new questions about that how lncRNAs function in different environments, which warrants additional studies in the future. As Zuo et al. claimed, in xenograft tumors of HCC, the overall survival rate in mice was significantly prolonged following treatment with a PLGA-based siLINC00958 nanoplatform (34). The polymeric nanoparticle platform is formulated with poly (lactic acid/glycolic) copolymer (PLGA) and can deliver different drugs including siRNAs. This nanotherapy is unlike conventional therapies and exhibits limited systemic toxicity, low immunogenicity, good controllability, high-efficiency, and tumor targeting. It is a promising approach to target disease mediated by the dysregulation of noncoding RNAs.

However, the study of m6A modification is still in its infancy, biological functions of the m6A-related protein family still need to be further explored. First, m6A modification can occur in most tumors, it is difficult to identify the specific m6A effectors in different tumors accurately. Second, there is no recognized reference method for the detection of m6A modification, methylated RNA immunoprecipitation combined with high throughput sequencing (MeRIP-Seq) can only identify the m6A hypermethylated region, but cannot quantitatively analyze m6A level or detect single-based m6A methylation. Third, the crosslink between m6A modification and noncoding RNAs is complicated, the specific binding sites between m6A methylation and ncRNA need further study. Potential biomarkers for diagnosis and adjuvant therapies will come from a better understanding of these action mechanisms.

## 7 Conclusion

The aberrant levels of m6A have been associated with multiple activities of ncRNAs, such as lncRNA metabolism, miRNA biogenesis, and circRNA translation, especially in eukaryotic organisms. Accumulating evidence has suggested that dysregulation of m6A modification leads to multiple diseases including carcinomas. Therein, to a great extent, the complicated regulatory mechanisms involved remain uncharted territory. In this review, based on the current literature, we have described the crosstalk between m6A deposition and ncRNAs on determining cell development and fate. m6A-derived transcriptome topology broadens our horizons into the mechanisms underlying gene expression. Further studies are necessary to reveal the biochemical and structural basis of these ncRNAs and m6A modifications, to design selective inhibitors and potential therapies in the future.

## Author Contributions

All authors listed have made a substantial, direct, and intellectual contribution to the work and approved it for publication.

## Conflict of Interest

The authors declare that the research was conducted in the absence of any commercial or financial relationships that could be construed as a potential conflict of interest.

## Publisher’s Note

All claims expressed in this article are solely those of the authors and do not necessarily represent those of their affiliated organizations, or those of the publisher, the editors and the reviewers. Any product that may be evaluated in this article, or claim that may be made by its manufacturer, is not guaranteed or endorsed by the publisher.

## Abbreviations

AGO2, Argonaut RISC catalytic component 2; ALKBH5, Alkylated DNA repair protein AlkB homolog 5; CDCP1, CUB domain containing protein 1; DGCR8, Di George syndrome critical region 8; eIF3, Eukaryotic translation initiation factor 3; EMT, Epithelial-mesenchymal transition; FAM225A, Family with sequence similarity 225 member A; FMR1, Fragile X mental retardation protein translational regulator 1; FOXM1, Forkhead box M1; FTO, Fat mass and obesity associated protein; GAS5, Growth arrest special 5; HAKAI, Also known as CBLL1, Cbl protooncogene like 1; HBXIP, Hepatitis BX-interacting protein; HDGF, Hepatoma-derived growth factor; HNRNP, Heterogeneous nuclear ribonucleoprotein; HNRNPA2/B1, Heterogeneous nuclear ribonucleoprotein A2/B1; IGF2BP1/2/3, Insulin-like growth factor 2 mRNA-binding protein1/2/3; IRES, Internal ribosome entry site; ITGB3, Integrin subunit beta 3; KCNK15-AS1:KCNK15 and WISP2 antisense RNA 1; MALAT1, Metastasis-associated lung adenocarcinoma transcript 1, also known as NEAT2; MAPK, Mitogen activated kinase-like protein; MEG3, Maternally expressed 3; mESC, Mouse embryonic stem cells; METTL14, Methyltransferase like 14; METTL3, Methyltransferase like 3, also known as MT-A70; NEAT1, Nuclear paraspeckle assembly transcript 1; NKAP, NFKB activating protein; ORF, Open reading Frame; PCAT6, Prostate cancer associated transcript 6; PVT1, Plasmacytoma variant translocation 1; RBM15, RNA binding motif protein 15; VIRMA, Vir-like m6A methyltransferase associated, also known as KIAA1429; WTAP, Wilms tumor 1-associating protein; XIST, X-inactive specific transcript; YAP, Yes-associated protein; YTHDC1, YTH domain containing 1; YTHDF1, YTH N6-methyladenosine RNA binding protein 1; ZC3H13, Zinc finger CCCH-type containing 13; TRIM25, tripartite motif containing 25; FAAH, fatty acid amide hydrolase.
